# Supplementary material for: Comparative genomics provides new insights into the diversity, physiology, and sexuality of the only industrially exploited tremellomycete: Phaffia rhodozyma
Source: BMC Genomics. 2016 Nov 9;17:901. doi: 10.1186/s12864-016-3244-7 (PMC5103461; doi:10.1186/s12864-016-3244-7)
Supplement: Additional file 6: — List of orphan genes with links to PFAM (related to Additional file 1: Table S1). (ZIP 1428 kb) [file 12864_2016_3244_MOESM6_ESM.zip › BLAST_HTML_FTR/G04061_P.html]

BLAST Search Results


```
BLASTP 2.2.27+


Reference:
Stephen F. Altschul, Thomas L. Madden, Alejandro A. Schäffer,
Jinghui Zhang, Zheng Zhang, Webb Miller, and David J. Lipman (1997),
"Gapped BLAST and PSI-BLAST: a new generation of protein database
search programs", Nucleic Acids Res. 25:3389-3402.


Reference for
composition-based statistics:
Alejandro A. Schäffer, L. Aravind, Thomas L. Madden, Sergei
Shavirin, John L. Spouge, Yuri I. Wolf, Eugene V. Koonin, and
Stephen F. Altschul (2001), "Improving the accuracy of PSI-BLAST
protein database searches with composition-based statistics and
other refinements", Nucleic Acids Res. 29:2994-3005.


Database: nr
           71,551,133 sequences; 26,053,659,533 total letters


Query= G04061_P

Length=235
                                                                      Score     E
Sequences producing significant alignments:                          (Bits)  Value

emb|CED84820.1|  hypothetical protein [Xanthophyllomyces dendrorh...   469    2e-165
gb|KOP41072.1|  pH-response transcription factor [Madurella mycet...  39.3    2.6   
ref|WP_018590858.1|  PAS domain-containing two-component system s...  39.3    3.5   
ref|XP_009951096.1|  PREDICTED: LOW QUALITY PROTEIN: uncharacteri...  38.5    6.9   


 >emb|CED84820.1| hypothetical protein [Xanthophyllomyces dendrorhous]
Length=234

 Score =  469 bits (1206),  Expect = 2e-165, Method: Compositional matrix adjust.
 Identities = 234/234 (100%), Positives = 234/234 (100%), Gaps = 0/234 (0%)

Query  1    MFHTQPNSHGGSQRLIALSHQKLEYLSYLPVDAPVLLRSVLIFNAIQVAESAGNAQRLRE  60
            MFHTQPNSHGGSQRLIALSHQKLEYLSYLPVDAPVLLRSVLIFNAIQVAESAGNAQRLRE
Sbjct  1    MFHTQPNSHGGSQRLIALSHQKLEYLSYLPVDAPVLLRSVLIFNAIQVAESAGNAQRLRE  60

Query  61   EEWFENTLGQLDSDEGDVSEIAPGSMSVPSPASAHSSESSAIVQVQEVDDDDDESDLDDE  120
            EEWFENTLGQLDSDEGDVSEIAPGSMSVPSPASAHSSESSAIVQVQEVDDDDDESDLDDE
Sbjct  61   EEWFENTLGQLDSDEGDVSEIAPGSMSVPSPASAHSSESSAIVQVQEVDDDDDESDLDDE  120

Query  121  EMSSLTGSVDSLLSFTMEDTEHNHAYPTTSPPPSRPASPRHSPPPQLFLPLPVAKRPTSI  180
            EMSSLTGSVDSLLSFTMEDTEHNHAYPTTSPPPSRPASPRHSPPPQLFLPLPVAKRPTSI
Sbjct  121  EMSSLTGSVDSLLSFTMEDTEHNHAYPTTSPPPSRPASPRHSPPPQLFLPLPVAKRPTSI  180

Query  181  VPFDDLSDDDDGPELVTPPQPALLDDFDTPCDGHSTDDFGWNAERATQGLQLFI  234
            VPFDDLSDDDDGPELVTPPQPALLDDFDTPCDGHSTDDFGWNAERATQGLQLFI
Sbjct  181  VPFDDLSDDDDGPELVTPPQPALLDDFDTPCDGHSTDDFGWNAERATQGLQLFI  234


>gb|KOP41072.1| pH-response transcription factor [Madurella mycetomatis]
Length=395

 Score = 39.3 bits (90),  Expect = 2.6, Method: Compositional matrix adjust.
 Identities = 36/126 (29%), Positives = 56/126 (44%), Gaps = 24/126 (19%)

Query  73   SDEGDVSEIAPGSMSVPSPASAHSSESSAIVQVQEVDDDDDESDLDDEEMSSLTGSVDSL  132
            SDEG      PG M+ P PA+          + Q+ +DD+    LD+ E   +   + +L
Sbjct  9    SDEGR-----PGYMATPVPAAE---------KYQKYEDDEKHRRLDEGEAMDVDLPLSNL  54

Query  133  LSFTME--DTEHNHAYPTTSPPPSRPASPRHSPPPQLFLPLPVAKRPTSIVPFDDLSDDD  190
              + ME  DT   +  P T+  P RP+        Q+ + L  ++R     P D+LSD +
Sbjct  55   HKYHMEGRDTTSRNRSPGTAATPGRPS--------QVAVVLHSSQRRDGFTPVDELSDGE  106

Query  191  DGPELV  196
                LV
Sbjct  107  GQSRLV  112


>ref|WP_018590858.1| PAS domain-containing two-component system sensor histidine kinase 
[Terrisporobacter glycolicus]
Length=798

 Score = 39.3 bits (90),  Expect = 3.5, Method: Composition-based stats.
 Identities = 19/80 (24%), Positives = 40/80 (50%), Gaps = 0/80 (0%)

Query  60   EEEWFENTLGQLDSDEGDVSEIAPGSMSVPSPASAHSSESSAIVQVQEVDDDDDESDLDD  119
            E++W+ NTLG +  +  +V  IA   + +      + +   A+ Q+ ++  D    D+D 
Sbjct  194  EKKWYSNTLGPIFDESNNVKYIASCKLDITLTKFLNDTLQIALNQITQISSDISIFDMDK  253

Query  120  EEMSSLTGSVDSLLSFTMED  139
            E  S +  +++ L+ F+  D
Sbjct  254  EYKSLIQYTIEKLMKFSKSD  273


>ref|XP_009951096.1| PREDICTED: LOW QUALITY PROTEIN: uncharacterized protein KIAA0556 
homolog [Leptosomus discolor]
Length=1578

 Score = 38.5 bits (88),  Expect = 6.9, Method: Compositional matrix adjust.
 Identities = 35/134 (26%), Positives = 62/134 (46%), Gaps = 12/134 (9%)

Query  31   VDAPVLL--RSVLIFNAIQVAESAGNAQRLREEEWFENTLGQLDSDEGDVSEIAPGSMSV  88
            V+AP +   ++ L F  +Q + ++ N + L EE   E  +  +   +  +SE+A     +
Sbjct  592  VNAPAVTEKKADLHFKCLQSSNTSLNWKSLPEENLLEQKMNSVSFTKKKLSELAGDLKVL  651

Query  89   PSPASAHSSESSAIVQV-QEVDDDDDESDLDDEEMSSL--------TGSVDSLLSFTMED  139
            PS      ++ +A  Q  +E D  DDE  L  E+M  +        TG++ S L  + + 
Sbjct  652  PSQVCMKDAKENATAQAAKEHDQSDDELTLS-EQMEKVTGRKFSDSTGAIPSWLQSSSQV  710

Query  140  TEHNHAYPTTSPPP  153
            TE+N    +   PP
Sbjct  711  TENNQVTSSKQKPP  724


Lambda      K        H        a         alpha
   0.314    0.132    0.391    0.792     4.96 

Gapped
Lambda      K        H        a         alpha    sigma
   0.267   0.0410    0.140     1.90     42.6     43.6 

Effective search space used: 1389040276235


  Database: nr
    Posted date:  Sep 23, 2015 12:05 AM
  Number of letters in database: 26,053,659,533
  Number of sequences in database:  71,551,133


Matrix: BLOSUM62
Gap Penalties: Existence: 11, Extension: 1
Neighboring words threshold: 11
Window for multiple hits: 40
```
